# Development of LCEs with 100% Azobenzene Moieties: Thermo-Mechanical Phenomena and Behaviors

**DOI:** 10.3390/mi13101665

**Published:** 2022-10-03

**Authors:** Domenico Sagnelli, Massimo Rippa, Amalia D’Avino, Ambra Vestri, Valentina Marchesano, Lucia Petti

**Affiliations:** Institute of Applied Sciences and Intelligent Systems of CNR, 80072 Pozzuoli, Italy

**Keywords:** azobenzene, photo-mobile polymers, liquid crystals, thermal properties

## Abstract

Azobenzene is one of the most investigated photo-responsive liquid crystalline molecules. It can isomerize between two different isoforms, trans (E) and cis (Z) configurations, when stimulated by light. It is used as a molecular engine in photo-mobile materials (PMPs). The use of liquid crystals (LCs) as building blocks enhances the mechanical properties of the PMPs. It is not easy to obtain PMPs with monodomain configurations when the LCs are 100% azobenzene. In this work, we studied three LC mixtures, describing the thermo/mechanical phenomena that regulate the actuation of such materials. The nematic temperature of the LC elastomers was measured and the PMPs carefully characterized for their bending and speed capability. Our finding suggests that the ratio between linear and cross-linker monomer greatly influences the nematic temperature of the mixture. Furthermore, 100% azobenzene materials polymerized using dicumyl peroxide can be useful to design polarization-selective switches.

## 1. Introduction

Conversion of light into mechanical work has been a hot topic for over 50 years. This approach is of great interest because actuators can be temporally, spatially, and remotely controlled by light, which can be beneficial in various applications [1,2,3,4,5,6,7].

One of the most investigated photo-responsive liquid crystalline molecules is the azobenzene [5,8,9,10,11] that isomerizes between trans (E) and cis (Z) configurations [12,13] when stimulated by polarized light. This isomerization can be exploited to use azobenzene as an engine to impart phototropic properties to materials. Azobenzene has been included in materials or as a dopant dye or as moiety in the polymeric network. In 1967, Lovrien [14] was the first to dope a polymer with photosensitive chromophores (such as azobenzene) generating a photomechanical outcome. However, phase separation of the chromophore and the matrix might occur, which is the major drawback. Initially in the early works [15], the synthesis of polymer networks with embedded azobenzene had a limited contractile strain (less than 1%) and the materials used to record optical gratings [16,17]. Later, with the use of liquid crystal (LCs)-based acrylate monomers, it was possible to improve the strength and strain of such materials [18,19]. This approach is of interest, since the characteristic anisotropy of LCs increases the mechanical properties of the polymer. The use of azobenzene molecules embedded as moieties in liquid crystal elastomers (LCEs) yields photo-mobile materials that efficiently convert light into mechanical output [5,19]. Subsequently, the focus in the literature was to decrease their concentration to allow the use of photo-initiators, which are greener and easier to use compared to traditional initiators. Photo-initiators cannot absorb enough energy to start polymerization in the presence of an azobenzene concentration higher than 20%. Furthermore, it is difficult to obtain PMPs with monodomain configurations with 100% azobenzene, since the nematic temperature range of azo-based LCs is quite narrow. In this work, three LC mixtures comprised of 100% of azobenzene moieties were developed and characterized. In particular, the thermo/mechanical phenomena that regulate the actuation of such materials are described. Also, as compared to traditional methods [5,6], the polymerization was achieved using dicumyl peroxide. Our finding suggests that such material can be useful for designing polarization-selective switches. The polarization effect on the activity of the switch seems to be independent from thermotropic components.

## 2. Materials and Methods

### 2.1. Preparation of Cell Reactor

For preparing reactor cells, we used glass slides and plastic spacers. Each glass slide was cut at needed measurements using a diamond tip. Subsequently, they were washed using, in sequence, first, glassware liquid cleaner (10 min sonic bath), then ultra-pure water (10 min sonic bath), acetone (20 min sonic bath), and finally isopropanol (5 min sonic bath). Once dry, the glass slides were spin-coated (3 s 4000 rpm, 30 s 4000 rpm, 3 s 0 rpm) using Elvamide (1%, 3%, 6%, *w*/*w* concentration in methanol). Finally, the slides were rubbed automatically using an automatized home-made machine. The rubbed sides of two glasses were faced anti-parallelly (respect to the rubbed direction) and spaced with a 50 µm thick Kapton layer to obtain a cell reactor.

### 2.2. Themo-Optical Characterization of Monomer Mixtures

To evaluate the orientational order of the mixture of LC monomers, we estimated the order parameter using linear dichroism measurements by transmission of light through the sample, with a set-up composed of: polarized laser (wavelength 633 or 457 nm), a heater chamber with an optical window (Mettler Toledo FP82HT), and a power detector (Coherent Model M-2). To evaluate the order parameter from polarized transmitted light, we used the dichroic ratio as reported in Equation (1) [10], with a transition moment parallel to the principal axis of the molecules:

(1) $$S=\frac{\left(A∥-A⊥\right)}{\left(A∥+2A⊥\right)}\;$$

where A⊥ and AII are the absorbances of the cell containing the molten monomers mix, which have a director (defined as the mean value of the directions of the molecular long axes) *n* perpendicular or parallel, respectively, to the direction of the laser polarization. The absorbance was calculated as: $\log_{10}\frac{1}{T}$, where T represents the cell transmittance.

The birefringence of the molten mix was investigated by introducing a polarizer before the power detector, and measuring the transmittance when the cell was perpendicular, parallel, or tilted 45 degrees as compared to the direction of the laser polarization.

### 2.3. Synthesis of the Photo-Mobile Polymer

The PMP films were prepared using three mixtures of LC monomers. We used three different molar ratios between A6zA6 (cross-linker) and A6z2 (linear) (BEAM co. www.beamco.com–structures in Supplementary Materials Figure S3) of 1:8, 1:1, and 8:1. The initiator used was the dicumyl peroxide, and this was 6.6 mol% of the total. The mixture of LCs used in this work are achiral and nematic molecules.

The mixtures were solubilized in DCM to help the homogenization at room temperature. The solvent was left to evaporate overnight under the fume hood.

Once dry, the mixture was injected by capillarity in the cells and the initiator activated. Subsequently, the cells were heated to the nematic temperature using the heating stage of Mettler Toledo FP82HT.

### 2.4. Spectrophotometry Measurement

Spectrophotometer JASCO V-650 (accuracy 0.5 nm, range 190–850 nm, Oklahoma, OK, USA) was used to investigate the optical properties of the PMP films. Both total transmittance T (%) and total reflectance R (%) were measured using the integrating sphere JASCO ISN-722 (inside diameter 60 mm, range 200–870 nm).

### 2.5. Photo-Actuation Characterization of PMPs

The PMPs actuators were cut as cantilevers (5 mm × 1 mm) and irradiated at 405, 457, 532, and 785 nm with 100:1 polarized laser. The set-up was composed of a neutral density filter, a retarder waveplate (λ/2), a focusing lens, and a sample holder mounted on a 3D translator.

The movements of the cantilevers were recorded at 60 fps. The movies were unpacked using virtualDub (v1.10.4). The bending angle was measured from the frames collected, considering both the initial and the final position of the cantilever respective to a graph paper placed behind the sample. The angle was converted first in radiant then in arclength (mm), and the speed calculated in m/s. The seconds were obtained by considering the number of frames in which the cantilever reaches its maximum bending.

### 2.6. Thermographic Measurements

Thermal response of the PMP film under laser irradiation was investigated by thermographic measurements. Analysis was performed using a LWIR AVIO TVS500 camera with an uncooled microbolometric detector (spectral range 8–14 μm, FPA 320 × 240 pixels and NETD ~60 mK at 25 °C) mounted with a 22 mm focal lens with iFOV 1.68 mrad. Thermal movies of the PMP films with a frame rate of 10 Hz were recorded while they were irradiated with a laser wavelength of 457 nm and power in the range 10–80 mW. The commercial software, IRT Analyzer (GRAYESS Inc.), which was supplied with the camera, was used for monitoring the temperature in real-time and for basic operations.

### 2.7. Polarized Optical Microscopy

The experiments were performed with a polarized upright microscope (Olympus BX51).

## 3. Results and Discussion

### 3.1. Fabrication and Synthesis Optimization

In the first step of this work, the possibility of preparing the reactor cells with Elvamide-coated glasses was investigated. Three different concentrated solutions of Elvamide were studied for this purpose (1%, 3%, or 6% *w*/*w*). After the spin-coating, the Elvamide films were characterised with different thicknesses to understand their influence on the rubbing. In particular, with 1% solution, the thickness of the spin-coated layer is 25 nm, and such a low level of thickness does not allow the formation of proper rubbing paths; in fact, the PMPs prepared do not bend properly (Figure S1 in Supplementary Materials). When the concentration of Elvamide is increased up to 3% and 6%, the thickness is between 75 and 200 nm (Table 1). The best results in terms of alignment are obtained with a 6% Elvamide coating. In fact, the PMPs produced at 6% are optimal in term of bending (Figure S1 in Supplementary Materials).

Afterwards, the polymerization temperatures were investigated to obtain optimal PMPs using A6zA6 and A6z2 mixtures (A6zA6:A6z2 = 8:1 or 1:1 or 1:8 molar ratio).

**Figure 1:**
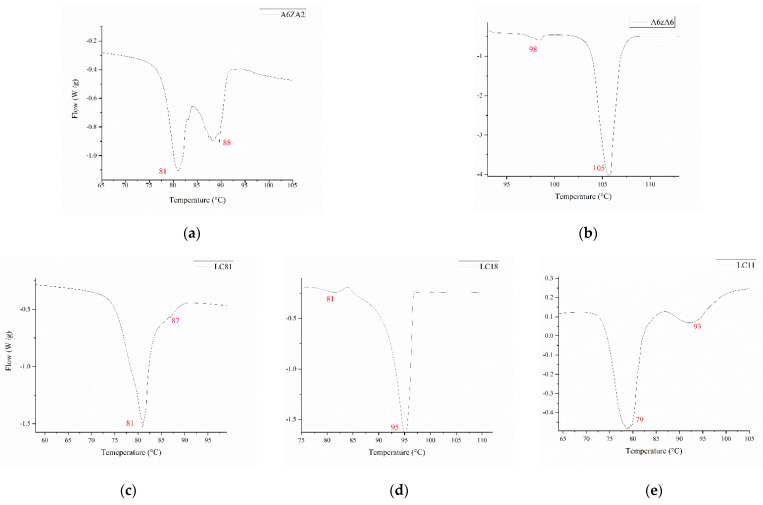
Melting profiles of LCs employed: (**a**,**b**) show the melting profile of A6zA2 and A6zA6, respectively, these are the monomer LCs used for the mixtures. (**c**) Melting profile of LC81 that does not show any nematic range, possibly because it is covered by the isotropic temperature. (**d**) Melting profile of LC18: in this case, again the isotropic temperature covers the nematic range. (**e**) Melting profile of LC11: in this case, it is possible to see the nematic phase between the two melting temperatures, indicating a range centered around 84 °C.

**Figure 2:**
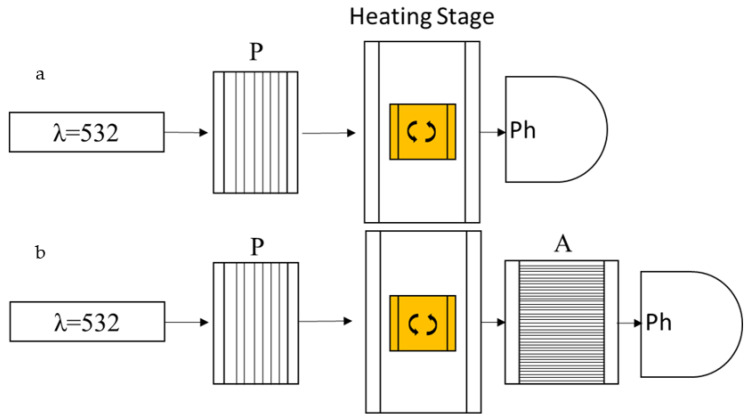
Schematic configuration of the transmittance measurement experiment. (**a**) Configuration to measure the state of order adjusting the position of the reactor cell parallel or orthogonal to the table. (**b**) Crossed polarized configuration to measure the transmittance when the rubbed cell is tilted 45 degrees as compared to the table. P and A are polarizer and analyzer, respectively. Ph is a photodiode used to measure the transmittance.

The first approach used to understand the nematic range of the mixtures in use was DSC (Figure 1). Unfortunately, such a technique does not show enough information to pinpoint a precise nematic temperature (Figure 1). In fact, either the nematic region is too short, meaning it is covered by the isotropic melting signal, or too large to select a single temperature. For these reasons, another approach was followed, as described in Figure 2.

This information was extrapolated from data collected using the set-ups described in Figure 2.

For the nematic phase, the order state can be defined as the preferred direction and a degree of order of the liquid crystals [20]. Such parameters can be used as a quantitative tool. Due to the opacity of our PMPs, we use them not as a quantitative method, but as a qualitative method, to narrow down the range of temperatures. For this reason, the S was used together with the transmittance of the LCs to pinpoint precisely the nematic temperature of the mixture.

In particular, the state of order of the mixtures was calculated as a function of the temperature. When the transition moment (defined as the electric dipole moment associated with the transition between the two isomers) is oriented parallel to the molecular long axis, the order parameter has the relationship (1) noted in *Materials and Methods*.

The transition moment is oriented parallel to the molecular long axis thanks to the rubbing of the cells, and subsequently parallel or perpendicular to the polarized light.

In particular, the cell reactors are infiltrated with the monomer mixtures in isotropic conditions (98–110 °C), in the absence of the initiator. Subsequently, the transmittance of the mixtures is measured between 75 and 100 °C with a rate of 0.5 °C/measure, using a 100:1 polarized laser with a wavelength of 532 nm (Figure 2a). The laser wavelength is chosen to avoid, as much as possible, the trans–cis isomerization of the azobenzene, and to have, at the same time, an appreciable absorbance. The transmitted light was measured with a power meter and converted in percentage, considering, as reference, the transmittance of an empty cell. Additionally, to evaluate the order state of the melted monomer mix, the measurements were performed with the director both parallel and orthogonal to the table. To precisely determine the polymerization temperatures, the maximum light-transmittance was measured with the cell between two crossed polarizers. Using this approach when the LC cell is rotated of 45 degrees in respect to the polarized light, and the LCs are nematic, the polarized light is rotated, and rotated again by the second polarizer, then having a maximum peak of transmission. In case the cell is in an isotropic state, or parallel to one of the two polarizers, the light is not rotated and cancelled by the second polarizer.

**Figure 3:**
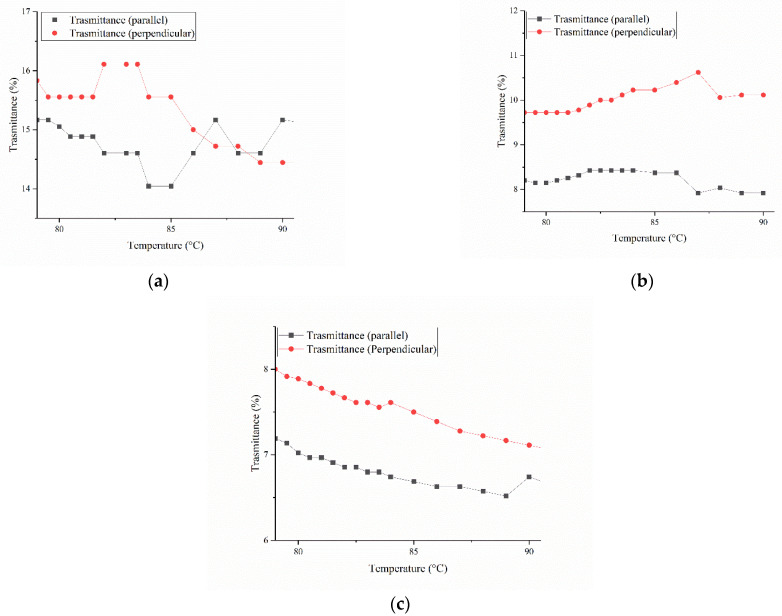
(**a**) Transmittance measurement of LC81. (**b**) Transmittance measurement of LC18. (**c**) Transmittance measurement of LC11.

These measurements were performed for all formulations to investigate the effect of the ratio between linear (L) and cross-linker (C) monomer on the LCs organization. In Figure 3a–c, we report the transmittance measurements for the three mixtures. First, the mix with a ratio of 8:1 (LC81) was measured. Interestingly, the transmission of light polarized parallelly and perpendicularly shows an opposite trend between 87.5 °C and 80 °C, indicating that in that range of temperatures, the mixtures of liquid crystals arrange from an isotropic to a nematic to a crystal phase. During the cooling, the transmittance signal decreases around 85 °C (Figure 3a) when the director is parallel to the laser polarization, showing that the molecules are recrystallizing from an isotropic state towards the nematic phase. For this reason, order state calculation was performed to pinpoint the current nematic temperature range. Consequently, the order state (S) calculations increase for the LCs in the range between 81.5 and 84 °C (Figure S2a in Supplementary Materials). To narrow this range down, an analyser was set parallel to the optical table between the heating stage and the power meter (Figure 2b). When the polarization of the laser and the axis of the analyser are perpendicular, the power meter shows a minimum in transmission, as long the nematic director of the cell is parallel to one of the two directions. When the cell is rotated 45 degrees instead at the isotropic–nematic transition, the maximum temperature in transmission is recorded. This happens because the liquid crystals induce a rotation to the polarization axis of the light. In fact, we found that only at 83, 82, and 81.5 °C does the mixture have a higher order state (Figure S2b in Supplementary Materials).

**Figure 4:**
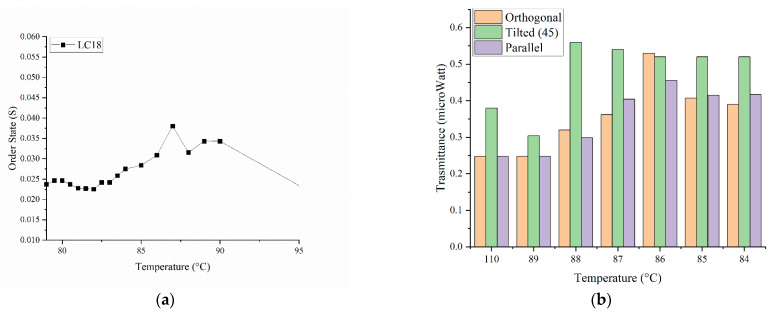
(**a**) Example of order state graph of LC18 and (**b**) transmittance of the cell between two crossed polarizers.

The same measurements were performed for the other mixtures. The mixture LC18 displays a flex in transmittance around 88 °C, possibly indicating that the passage from the isotropic to nematic phase starts there (Figure 3b). After this, we used the order parameter to narrow the temperature range (Figure 4a). In Figure 4a, it is possible to see when the LCs transition from isotropic to nematic. In fact, we clearly see this between 88 and 86 °C, when the signal rises from the baseline, indicating an increment in order. Again, to clarify the data obtained via S calculation, the transmittance of the cell was measured between two crossed polarizers. Using this approach, the nematic range is narrowed down to only two temperatures, 87 and 88 °C (Figure 4b). The last mixture (LC11) does not show any clear change in the transmittance graph (Figure 3c). When the order parameter is measured, a wide range of temperatures are found (Supplementary Figure S2c). Unfortunately, not even measuring the transmission between the two polarizers helps narrowing down the range (Figure S2d). At a molar ratio of 1:1, the two LCs possibly compete for the mix’s stabilization, never reaching an equilibrium. For these reasons, the temperature chosen for LC11 is based on the observation of the direct transmittance (Figure 3c), showing a slight peak at 84 °C.

### 3.2. Polymerization and Alignment Confirmation

The polymerization of monomer mixtures (A6Z2 and A6ZA6, Beamco; monomers were used as received) is achieved by injecting an initiator together with the mixture (Dicumyl peroxide-DPX-6.6 mol%) in the rubbed cells. The mixtures are infiltrated in an isotropic state (temperature ≥ 100 °C), avoiding the activation of the initiator (temperature > 110°). Traditionally, these type of monomers mixtures are polymerized with an initiator such azobisisobutyronitrile (AIBN), and infiltrated in a vacuum oven to remove bubbles [5]. DPX avoids the use of a vacuum oven; in fact, in the conditions used, gas bubbles do not form in the PMP. This approach greatly simplifies the synthesis of PMPs with 100% azobenzene moieties.

To obtain an isotropic configuration, the initiator was activated at 130 °C for 3 h and the sample kept at a temperature greater than 100 °C. To achieve a nematic organisation, the mixtures were kept at 160 °C for 20 s to initiate polymerization. Afterward, the PMP was placed on a stabilized heating plate or on a heating stage at the temperature measured previously.

**Figure 5:**
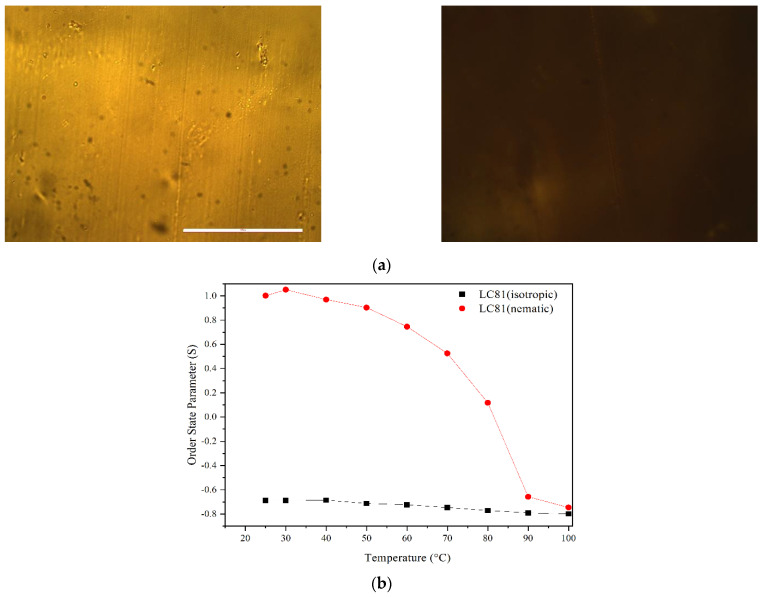
(**a**) Polarized microscopy images of LC18, showing the birefringence properties of the materials. The bar represents 200 um. (**b**) Order state parameter of LC81 measured at various temperatures (probe wavelength λ = 633 nm).

After the polymerization occurred, a polarized microscope was used to check the birefringence properties of the PMPs. Interestingly, the samples LC11 and LC18 are semi-transparent, and it is possible to demonstrate their ability to rotate the polarized light (Figure 5a). In contrast, the PMP LC81 is opaque and does not have considerable transmittance to measure its birefringence.

For this reason, the alignment of the azobenzene moieties in LC81 was studied performing a transmittance experiment, as previously explained for the monomer mixtures.

In brief, the transmittivity of the film was measured between 25 and 100 °C using a polarized He/Neon laser with a wavelength of 633 nm, for which the absorbance of the azobenzene it is close to zero.

Interestingly, the nematic PMP (Figure 5b 8:1 ratio) shows a highly ordered state (S = 0.9) between 20 and 30 °C. After increasing the temperature, the order state parameter decreases in all samples measured, indicating a correlation between temperature and LCs order state. This experiment shows that when the PMPs are polymerized at a lower temperature (nematic temperature), the S of the azobenzene moieties is higher than when polymerized at higher temperatures (isotropic states). In fact, when the factor S of the films are compared, a clear difference between the two PMP is observed (Figure 4a). The PMPs polymerized at the LC nematic temperature (A6zA2:A6zA6 = 8:1) show a higher order state up to 70 °C. Instead, the PMP polymerized in isotropic state show a low order state starting from room temperature.

### 3.3. PMP Absorbance and Thermo-Mechanical Properties

**Figure 6:**
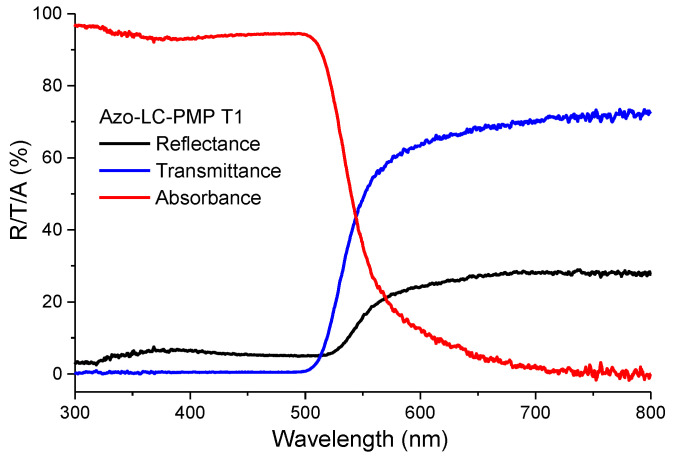
Spectral characterization of LC81: reflectance (black line), transmittance (blue line), and absorbance (red line).

The optical properties of PMPs were also investigated by spectrophotometric analysis. In Figure 6, the transmittance (blue line) and the reflectance (black line) measured for a PMP film with a thickness of 50 µm are reported. The absorbance of the film is successively calculated using the well-known relation A(%) = 100 − T(%) − R(%), and represented in the same figure with a red line. The absorbance estimated is higher than 90% in the wavelength range UV/VIS 200–500 nm, then the values decrease quickly to a few percentages in the near-infrared region (Figure 6). These results show how a laser source with wavelength of 457 nm can be an optimal choice to investigate the behavior of the film bending, because of the absorption of optical energy. Interestingly, the PMP shows reflectivity up to 30% after 550 nm. This effect is probably due to various factors. Polymeric material glossiness is related to its smoothness [21]. Probably, because of our preparation/polymerization procedure, the material does not show much reflectivity, since the rubbing causes the formation of a very rough surface. Another factor is correlated to the presence of azobenzene, which in our analysis condition, shows a light orange color. Azobenzene, even in different conditions than ours, was previously used to switch optical properties of liquid crystals mixtures and elastomers [22].

Subsequently, the three PMPs were characterized for their mechanical response to polarized light (457 nm). The samples were cut as cantilevers (5 × 1 mm) and positioned with the rubbing direction perpendicular to the optical table.

**Figure 7:**
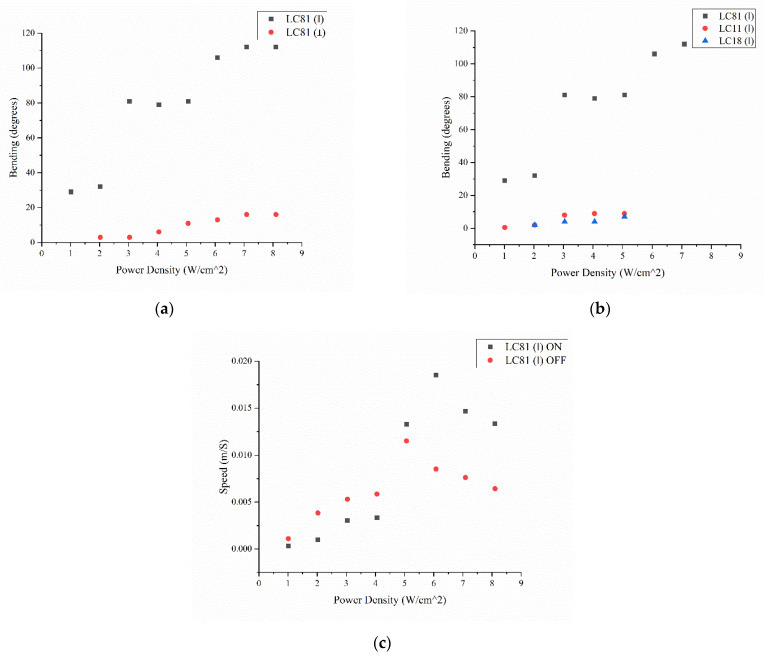
(**a**) Bending of LC81 when irradiated with polarized light parallel and perpendicular to the rubbing direction, (**b**) bending of LC81, LC18, and LC11 irradiated by polarized light parallel to the rubbing direction, and (**c**) bending speed of LC81 when the laser is ON and OFF.

First, the results show that the PMP LC81 is better at converting light into mechanical energy (Figure 7a). Second, the performances of the film is better when the laser polarization orientation is parallel to the rubbing direction compared to the perpendicular orientation.

For the first observation, it is obvious that the ability of the PMP LC81 to convert light into mechanical work is due to the degree of cross-linking. In fact, LC11 and LC18 have, respectively, four and eight times more the content of cross-linker. This results in more brittle and rigid materials.

The second observation is more difficult to justify, because it is deeply correlated to azobenzene properties. In fact, azobenzene behavior is correlated to the way it interacts with the polarized light. When the molecule long axis is parallel to the polarization of the laser, it absorbs the energy and uses it to isomerize to a more energetic isoform (trans>cis). Through its relaxation time (laser switched off) or heat, the molecule would back-isomerize to trans. This cycle happens a certain number of times (trans>cis>trans), until the molecular long axis of the azobenzene turns perpendicular to the polarization [23]. At that point, the molecule does not interact anymore with the laser [23]. That is clearly shown in Figure 7b, where the performance of the material is better when the orientation of the polarized light is parallel to the rubbing direction. This is more proof that our synthesis approach allows a good alignment and organization of LCs in the PMP.

LC81 is also characterized for its bending speed (up to 0.02 m/s), showing a fast and dose-dependent response to polarized light (Figure 7c). Interestingly, when the laser is turned off, the material returns to its starting position as fast as when bending with the light on.

**Figure 8:**
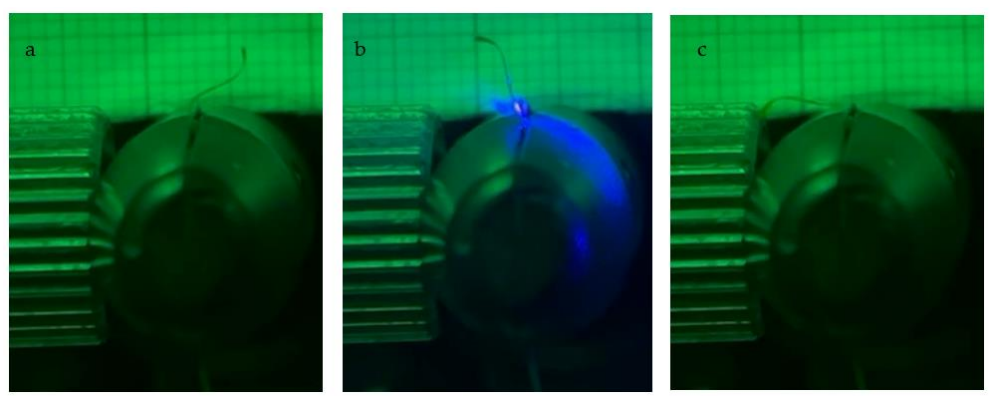
Movement modulation of the PMP as a function of the polarization. If the PMP is hit by a laser polarized parallel to the rubbing director, it bends toward the laser and then goes back to its initial position when the laser is turned off (**a**,**b**). When the PMP is firstly hit with a laser polarized perpendicularly and turned off and then hit with parallelly polarized light, the PMP changes bending direction (**c**).

This material is notably polarization-dependent, as already explained, but in this configuration, and at this specific polymerization temperature, we observe a new behavior (Figure 8). In fact, the PMP can be programmed to act as a switch using the polarization of the laser. If the PMP is hit by a laser polarized parallel to the rubbing director, it bends toward the laser and then goes back to its initial position when the laser is turned off (Figure 8a,b). When the PMP is firstly hit with a laser polarized perpendicularly and turned off and then hit with parallelly polarized light, the PMP changes bending direction (Figure 8c). Furthermore, this behavior can be reverted to the original by continuously irradiating the PMP first with parallel polarized laser, and then changing the polarization to perpendicular. In this case, the PMP reverts back to its initial configuration. This behavior is mainly observed with LC81, meaning that the degree of cross-linking plays a fundamental role in such behavior. Less cross-linking means a looser and more elastic network. Furthermore, the observed behavior, is probably correlated with the intrinsic nature of the materials. In fact, the PMPs studied are LCEs that notably retain the properties of their building blocks: liquid crystals. In this case, the sole liquid crystal used has azobenzene moieties that rotate its molecular long axis in function of the light polarization. For these reasons, we argue that the combination of azobenzene properties and the degree of cross-linking is the main cause of such behavior. Such behavior could possibly be finely modulated in depending on azobenzene concentration and degree of cross-linking.

**Figure 9:**
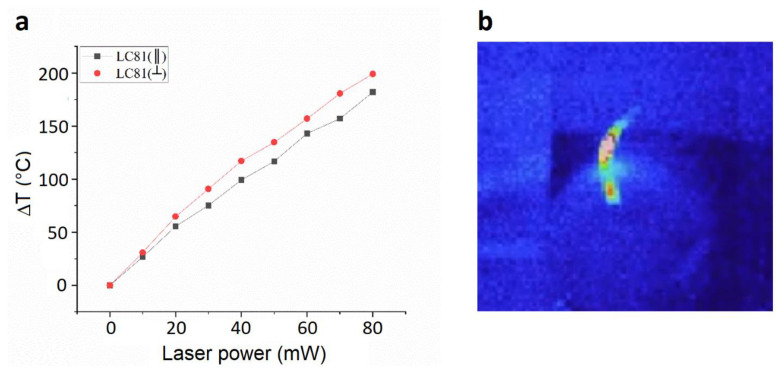
Thermographic analysis: (**a**) maximum temperature increases (ΔT) that the film reaches when irradiated with a laser wavelength of 457 nm with power in the range 0–80 mW, (**b**) example of thermal image acquired during the measurements.

Finally, we investigated the thermal response of the LC81 PMPs by thermographic analysis when irradiated with a laser wavelength of 457 nm. In Figure 9a, the maximum temperature increases (ΔT) that the film reaches in the range 0–80 mW are shown, considering both parallel and perpendicular laser polarization. As visible from the graph, the temperature trends achieved for the two different polarizations are similar, and can be considered quite linear with respect to the laser power. The temperature values with parallel polarization are only slightly higher than those measured with the perpendicular polarization. These results relate to the thermal behavior of the sample, and do not justify the differences in mechanical response (Figure 7b) associated with the two laser polarizations, indicating how the thermal effects do not play a predominant role in the film’s movement. In Figure 9b, an example of a thermal image acquired during the measurements is shown.

## 4. Conclusions

We developed and characterized three azobenzene-based photo-mobile polymer mixtures to understand how the ratio between the linear (L) and cross-linker (C) monomer influences their final properties. We found that the nematic temperature of the LCs mixture is greatly influenced by the ratio of L and C. In fact, increasing the molar ratio in favor of the cross-linker (LC18), considerably increases the nematic temperature (up to 88 °C).

Furthermore, we characterised their light-to-mechanical properties, and show that when the ratio is in favor of the linear monomer, the PMPs bend more and the process is faster compared to the other mixtures. Finally, we show that the thermal effects do not play a predominant role in the movement of the film.

## Figures and Tables

**Table 1:** Thickness of Elvamide coating layer before rubbing.

Name	Thickness
1% Elvamide	25 nm
3% Elvamide	75 nm
6% Elvamide	200 nm

## Data Availability

Not applicable.

## Supplementary Materials

The following supporting information can be downloaded at: https://www.mdpi.com/article/10.3390/mi13101665/s1, Figure S1: (Left) PMP polymerized in cells with 1% Elvamide. (Right) PMP polymerized in cells with 6% Elvamide. Figure S2: (**a**) order state of LC81, (**b**) transmittance of LC81 between crossed polarizers, (**c**) order state of LC11, (**d**) transmittance of LC11 between crossed polarizers. Figure S3: Here are depicted the LC monomers used in this work. The monomers are mixed in two different molar ratios (8:1 1:1 1:8). (Up) monomer A6zA6 (down) monomer A6z2.
