# Supplementary material for: Development of LCEs with 100% Azobenzene Moieties: Thermo-Mechanical Phenomena and Behaviors
Source: Micromachines (Basel). 2022 Oct 3;13(10):1665. doi: 10.3390/mi13101665 (PMC9607918; doi:10.3390/mi13101665)
Supplement: Supplementary file 1 [file micromachines-13-01665-s001.zip › micromachines-1877753-supplementary layout.pdf]

# Supplementary Materials: Development of LCEs with 100% Azobenzene Moieties: Thermo-Mechanical Phenomena and Behaviors

**Domenico Sagnelli *,†, Massimo Rippa †, Amalia D'Avino, Ambra Vestri, Valentina Marchesano and Lucia Petti**

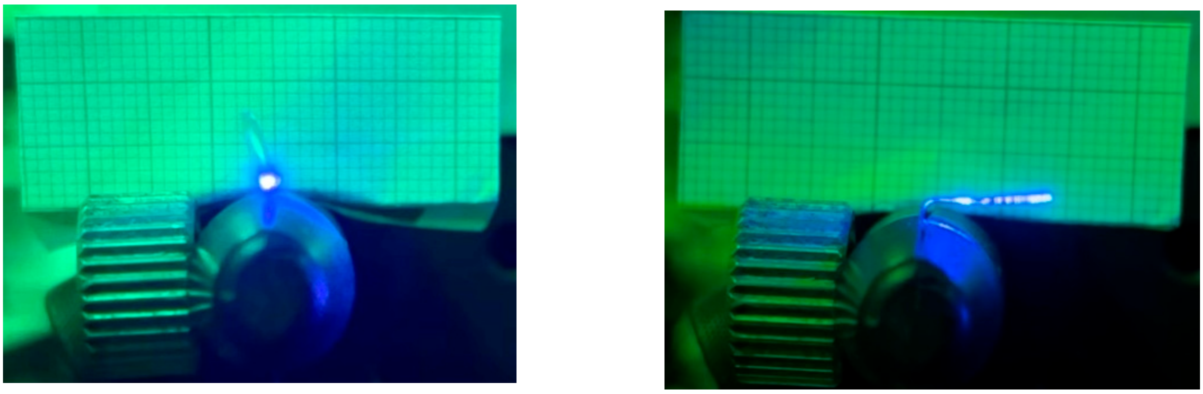

**Figure S1**. (**Left**) PMP polymerized in cells with 1% elvamide. (**Right**) PMP polymerized in cells with 6% elvamide.

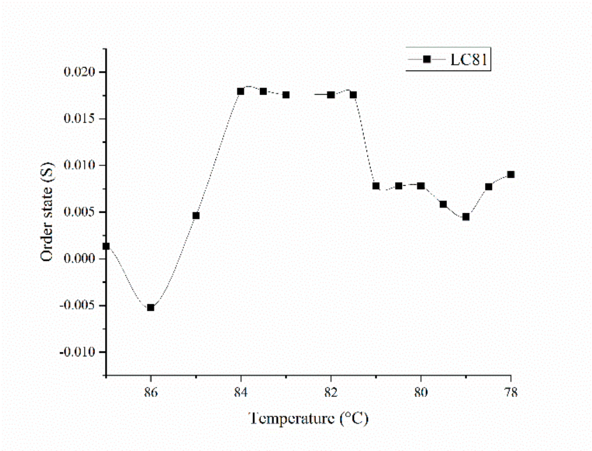


(a)

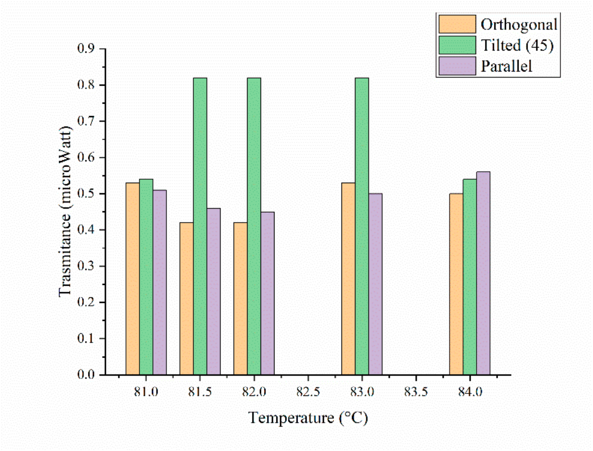


(b)

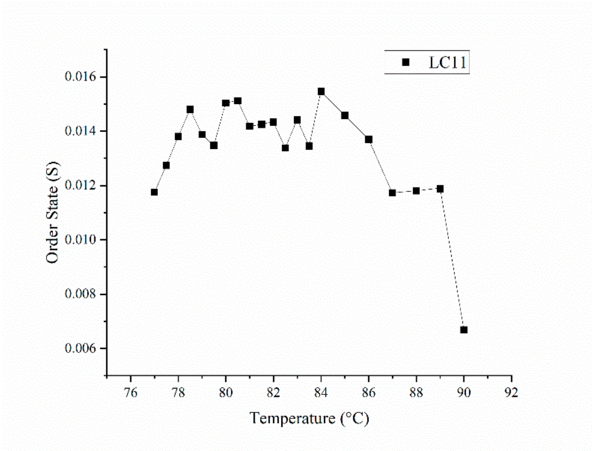


(c)

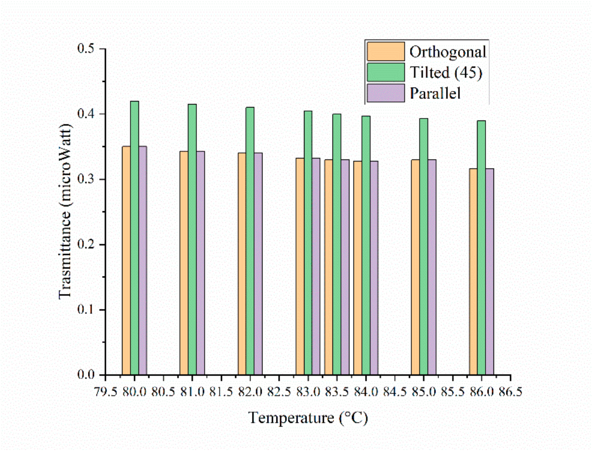


(d)

**Figure S2.** (**a**) Order State of LC81, (**b**) Transmittance of LC81 between crossed polarizers (**c**) Order state of LC11, (**d**) Transmittance of LC11 between crossed polarizers.

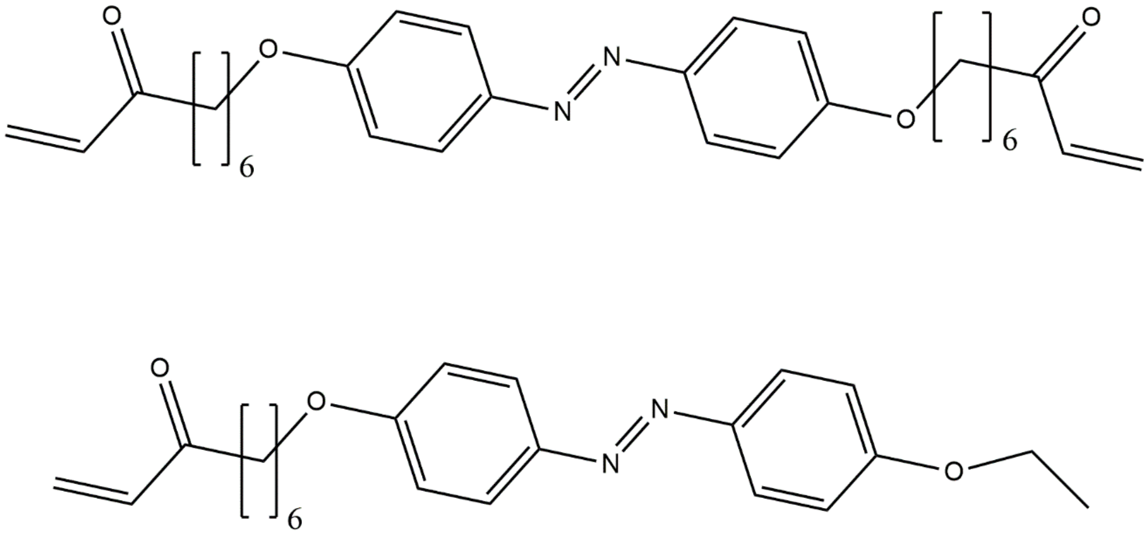

**Figure S3;-** Here are depicted the LC monomers used in this work. The monomers are mixed in two different molar ratios (8:1 1:1 1:8). Up) monomer A6zA6 down) monomer A6z2
